# Supplementary material for: Physical activity and sedentary behaviour counselling: Attitudes and practices of mental health professionals
Source: PLoS One. 2021 Jul 16;16(7):e0254684. doi: 10.1371/journal.pone.0254684 (PMC8284800; doi:10.1371/journal.pone.0254684)
Supplement: S1 Table — (DOCX) [file pone.0254684.s001.docx]

**S1 Table. Mental health professionals’ perceived importance of different types of treatment for people with mental illness**

| Treatment strategy | Percent of clinicians who ranked the strategy as… | | | |
| --- | --- | --- | --- | --- |
|  | the most important | | 2^nd^ most important | 3^rd^ most important |
| Cognitive behavioral therapy | 58.8% | 11.8% | | 11.8% |
| Social support | 41.2% | 11.8% | | 11.8% |
| Family therapy | 0% | 17.7% | | 29.4% |
| Vocational rehabilitation | 0% | 5.9% | | 5.9% |
| Medication | 0% | 23.5% | | 0% |
| Increasing physical activity | 0% | 29.4% | | 35.3% |
| Reducing sedentary behavior | 0% | 0% | | 17.7% |
| Social skills training | 0% | 0% | | 5.9% |
| Bright light therapy | 0% | 0% | | 0% |
| Electroconvulsive therapy | 0% | 0% | | 0% |
| Hospitalisation | 0% | 0% | | 0% |
