# Supplementary material for: Physical activity and sedentary behaviour counselling: Attitudes and practices of mental health professionals
Source: PLoS One. 2021 Jul 16;16(7):e0254684. doi: 10.1371/journal.pone.0254684 (PMC8284800; doi:10.1371/journal.pone.0254684)
Supplement: S2 Table — (DOCX) [file pone.0254684.s002.docx]

**S2 Table. Mental health professionals’ specific practices in recommending physical activity**

| **What methods do you use for recommending physical activity? *** | |
| --- | --- |
| Personal discussion | 88.2% |
| Brochures or pamphlets  Referral to community-based programs | 17.6%  35.3% |
| Referral to exercise professional | 17.6% |
| Nothing specific | 29.4% |
| Internet | 11.8% |
| Any sporting clubs they might be interested in | 5.9% |
| **How often do you recommend they engage in physical activity?** | |
| Every day | 17.6% |
| Most days of the week | 35.3% |
| Once to twice a week | 11.8% |
| As often as they can | 35.3% |
| **What intensity do you recommend they engage in activities? *** | |
| Low intensity | 11.8% |
| Moderate intensity | 23.5% |
| Vigorous intensity | 5.9% |
| At level that makes them feel good | 47% |
| I do not suggest intensity | 29.4% |
| **What type of physical activity do you recommend? *** | |
| Aerobic exercise | 82.4% |
| Weight training or resistance training | 41.2% |
| Swimming | 41.2% |
| Team sports | 64.7% |
| Combat sports | 35.3% |
| Dancing | 5.9% |
| Group sessions at gym | 5.9% |
| Any activity they enjoy | 29.4% |

*Multiple response option
