# Supplementary material for: Physical activity and sedentary behaviour counselling: Attitudes and practices of mental health professionals
Source: PLoS One. 2021 Jul 16;16(7):e0254684. doi: 10.1371/journal.pone.0254684 (PMC8284800; doi:10.1371/journal.pone.0254684)
Supplement: S1 File — (DOCX) [file pone.0254684.s003.docx]

**Focus group questionnaire**

1. What motivated you to participate in this trial?
2. What prevents you from participating in physical activity or interrupting the amount of time you spend sitting?
3. In your environment, what facilitates engaging in physical activity or sitting less? And what limits it?
4. What are your motivations to engage in a physical activity or conversely to not engage in a physical activity?
5. What are your motivations in sitting less?
6. Did anything encourage you to stand up more or walk to complete your work during the study period?
7. Did behavior of your colleagues encourage you (standing up or walking at work)?
8. Did the goals that you set encouraged you to stand up more or walk at work?
9. Did participation in this study increased the likelihood of you recommending physical activity to your clients?

a. How do you think your physical activity habits influence whether or not you engage in physical activity counseling with your clients?

b. How confident are you that you can counsel your clients about physical activity even if you are physically inactive?

1. Is physical activity part of your treatment approach?

PROBES: a. Who typically brings up the discussion about physical activity, you or your clients?

b. What kinds of questions about physical activity do your clients ask you?

c. What kind of questions about physical activity do you ask your clients?

d. Have you ever felt like you were unable to answer your client’s physical activity questions?

i. PROBE: What types of questions are you unable to answer? Any others? REPHRASE: Can you recall any other questions or times?

e. What do you do if you cannot answer?

PROBE: Do you find the answer for them? Where? How?

f. What would you tell your clients about why exercise is important for health?

1. Do you currently counsel your clients about their physical activity?

PROBES: a. How often do you counsel your clients about their physical activity levels? b. What are the characteristics or types of clients that you are more likely to counsel about physical activity?

c. What do you feel your strengths are when it comes to counseling physical activity, if any?

d. What do you feel your weaknesses are when it comes to counseling physical activity, if any?

1. Was there anything about using the intervention that you particularly liked?
2. In closing, is there anything else you’d like to say about your experience of participating in this study?
