# Supplementary material for: Physical activity and sedentary behaviour counselling: Attitudes and practices of mental health professionals
Source: PLoS One. 2021 Jul 16;16(7):e0254684. doi: 10.1371/journal.pone.0254684 (PMC8284800; doi:10.1371/journal.pone.0254684)
Supplement: Attachment — Submitted filename: PONE-20-32740-R1 Review Coment-AV Egwuonwu 11th June 2021.doc [file pone.0254684.s005.doc]

**PLOS ONE**

Physical activity and sedentary behaviour counselling: attitudes and practices of mental health professionals --Manuscript Draft- Reviewer’s Comments AV Egwuonwu 11^th^ June 2021.

**Acknowledgement of all corrections**

I endorse to the editors to publish this well written manuscript in PLOS ONE

Afamefuna Victor Egwuonwu (PhD) Expert in Physical Activity, Musculoskeletal Physiotherapy and Ergonomics
