# Supplementary material for: Physical activity and sedentary behaviour counselling: Attitudes and practices of mental health professionals
Source: PLoS One. 2021 Jul 16;16(7):e0254684. doi: 10.1371/journal.pone.0254684 (PMC8284800; doi:10.1371/journal.pone.0254684)
Supplement: Attachment — Submitted filename: PONE-D-20-32740_Response to reviewers 2.docx [file pone.0254684.s006.docx]

Dear Editors,

Thank you for your kind suggestions.

We have made the additional changes to the manuscript PONE-D-20-32740R1 that were recommended. Please find our point-by-point responses to your suggestions below.

Thanking you for your consideration.

Yours sincerely,

Authors

Response to Editor’s comments

Editor’s comment: Please provide justification for the sample size (or whether data satisfaction has been reached) and acknowledge the limitation of the small sample size of the study.

Authors’ response:

According to your suggestions, we have added the following sentences to the manuscript:

*Lines 163-165:*  *Each FGD was limited to five participants to ensure that the discussion is sufficiently interactive, inclusive, and participatory and that it allows participants to share in-depth insights in their attitudes and practices. The number of participants per FGD in our study was in accordance with the commonly accepted sample size recommendations for FGDs [29].*

*Lines 168-170:*  *In the second FGD, the themes that evolved overlapped sufficiently enough with the first FGD to assume an adequate saturation of data [30].*
